# Supplementary material for: Unveiling the Anti-Cancer Potential of Onoceranoid Triterpenes from Lansium domesticum Corr. cv. kokosan: An In Silico Study against Estrogen Receptor Alpha
Source: Int J Mol Sci. 2023 Oct 9;24(19):15033. doi: 10.3390/ijms241915033 (PMC10573215; doi:10.3390/ijms241915033)
Supplement: Supplementary file 1 [file ijms-24-15033-s001.zip › ijms-2600030-supplementary.pdf]

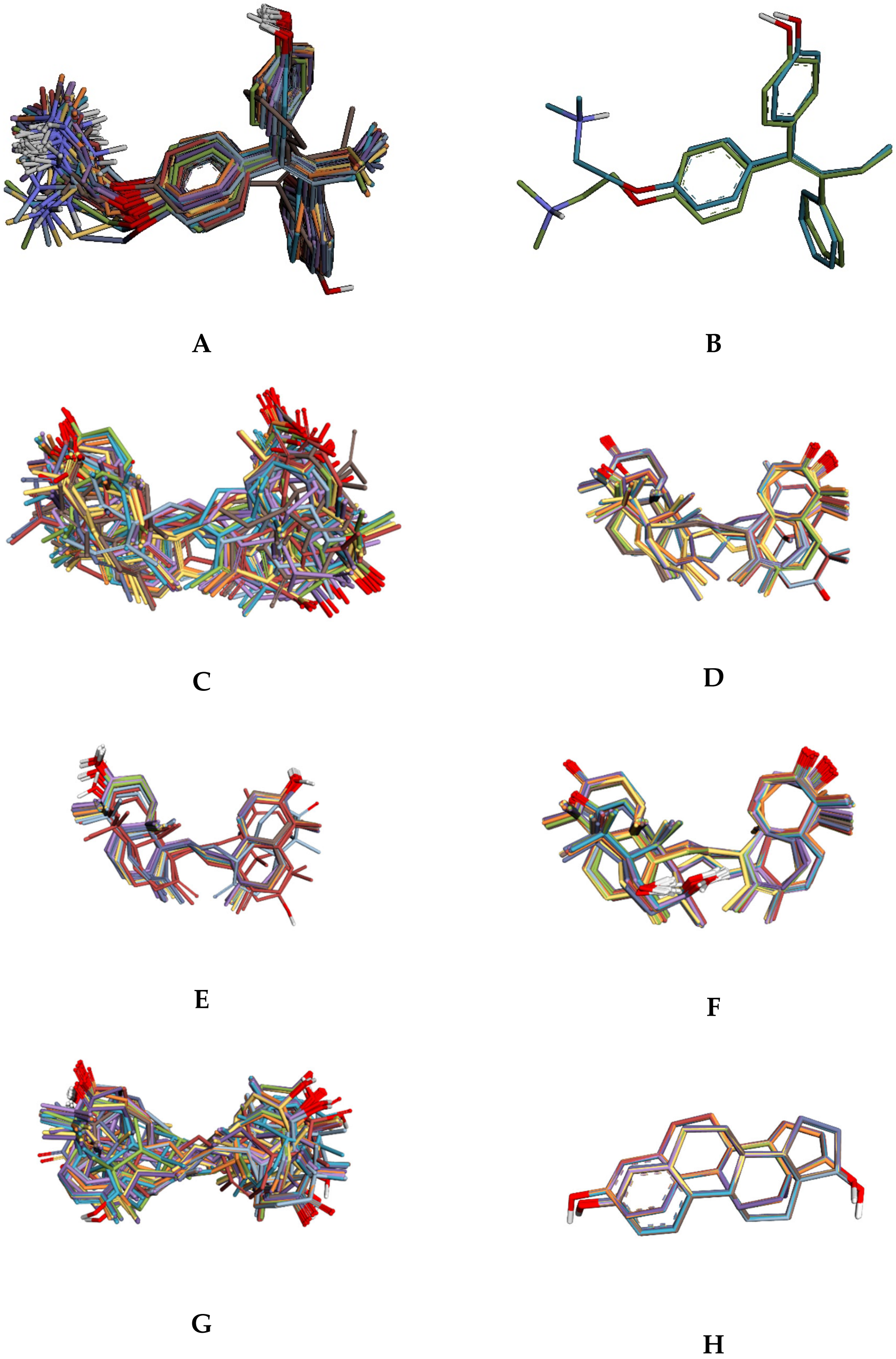

Figure S1. Redocking results of tamoxifen to ERα. (**A**) 100-Structure overlay of poses generated from tamoxifen redocking procedure. Ninety-nine poses reproduce the crystal structure conformation

with RMSD values below 1.50. (**B**) Overlay of the redocking pose with the best binding energy score (turquoise) and the crystal structures (green) of tamoxifen. First Cluster-Structure overlay of poses generated from (**C**) compound 1, (**D**) compound 2, (**E**) compounf 3, (**F**) compound 4, (**G**) compound 5, and (**H**) Estradiol docking procedures.

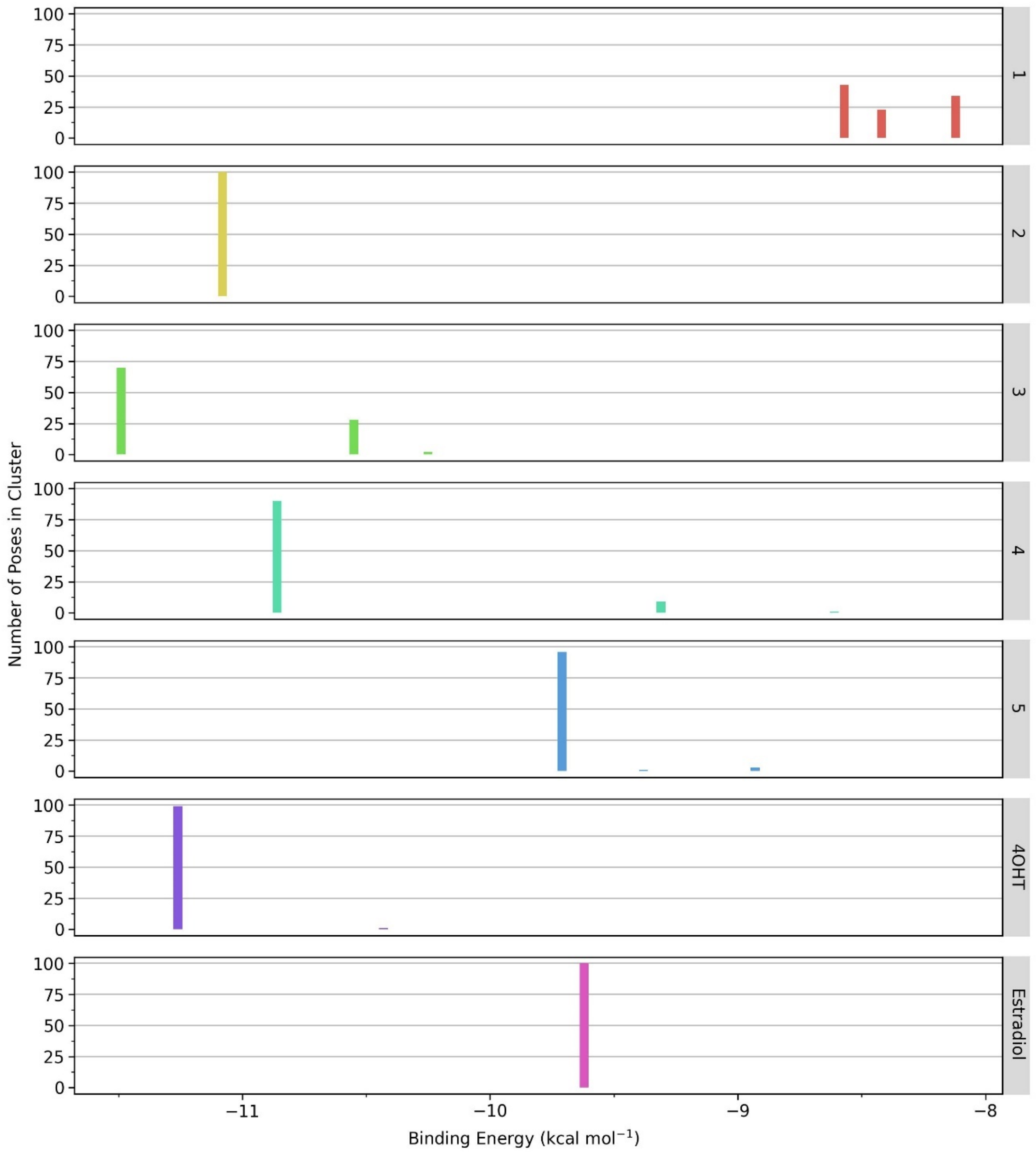

Figure S2. Cluster analyses of docking poses of **1**, **2**, **3**, **4**, **5**, 4OHT, and estradiol to ERα. The docking poses were generated by AutoDock4.2.

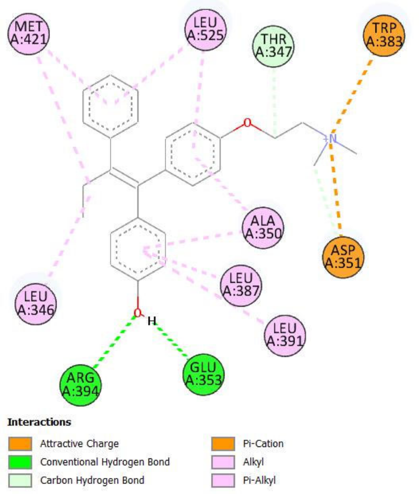

4OHT

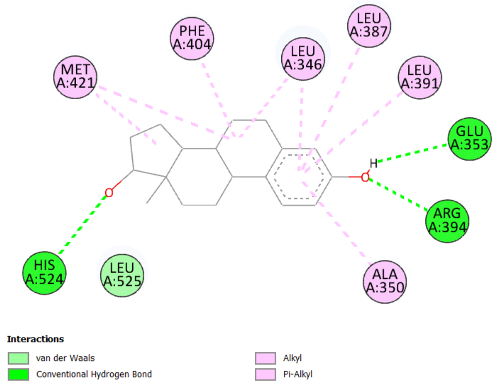

Estradiol

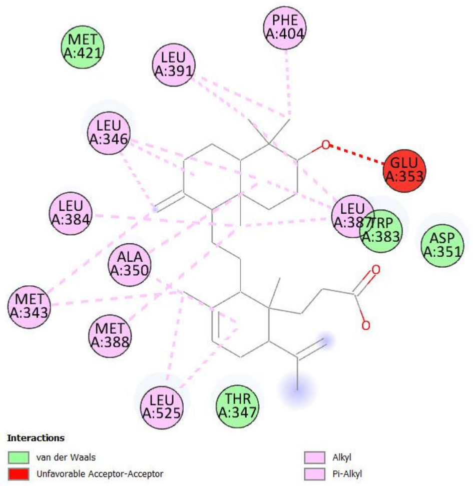

1

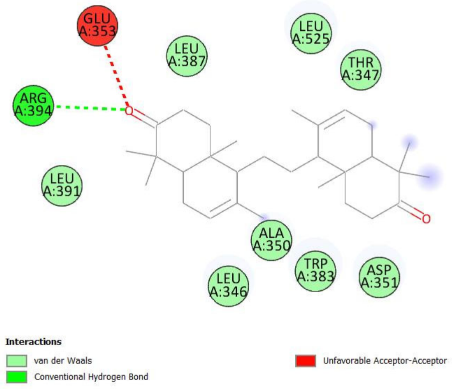

2

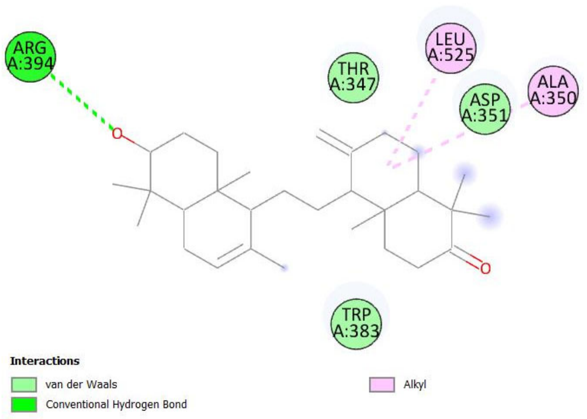

3

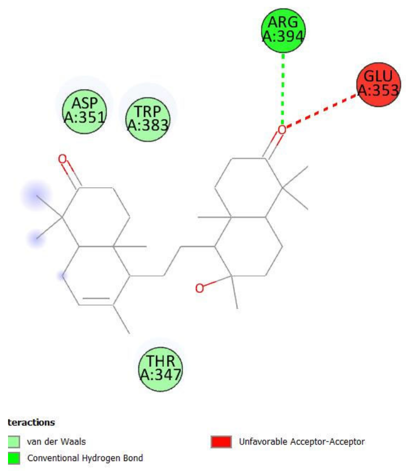

4

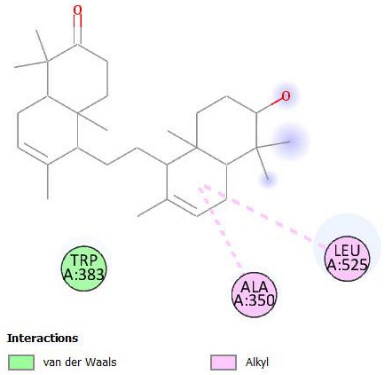

5

Figure S3. Molecular interactions between ligands and ERα. The ligands include **1**, **2**, **3**, **4**, **5**, 4OHT, and estradiol.

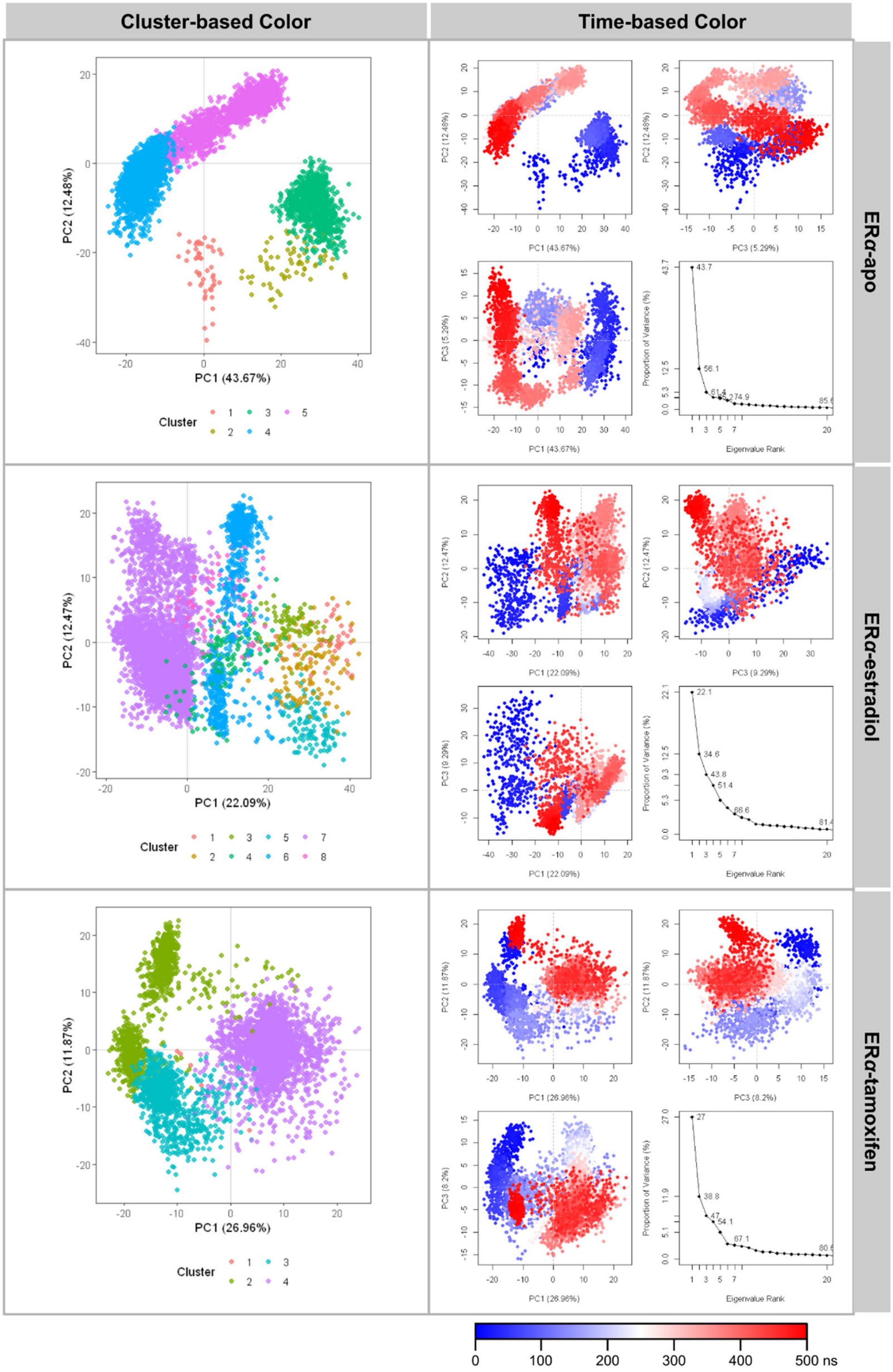

Cluster-based Color
Time-based Color
ERα-apo
ERα-estradiol
ERα-tamoxifen
PC1 (43.67%)
PC2 (12.48%)
PC3 (5.29%)
Cluster
PC1 (22.09%)
PC2 (12.47%)
PC3 (9.29%)
PC1 (26.96%)
PC2 (11.87%)
PC3 (8.2%)
Proportion of Variance (%)
Eigenvalue Rank
0
100
200
300
400
500 ns

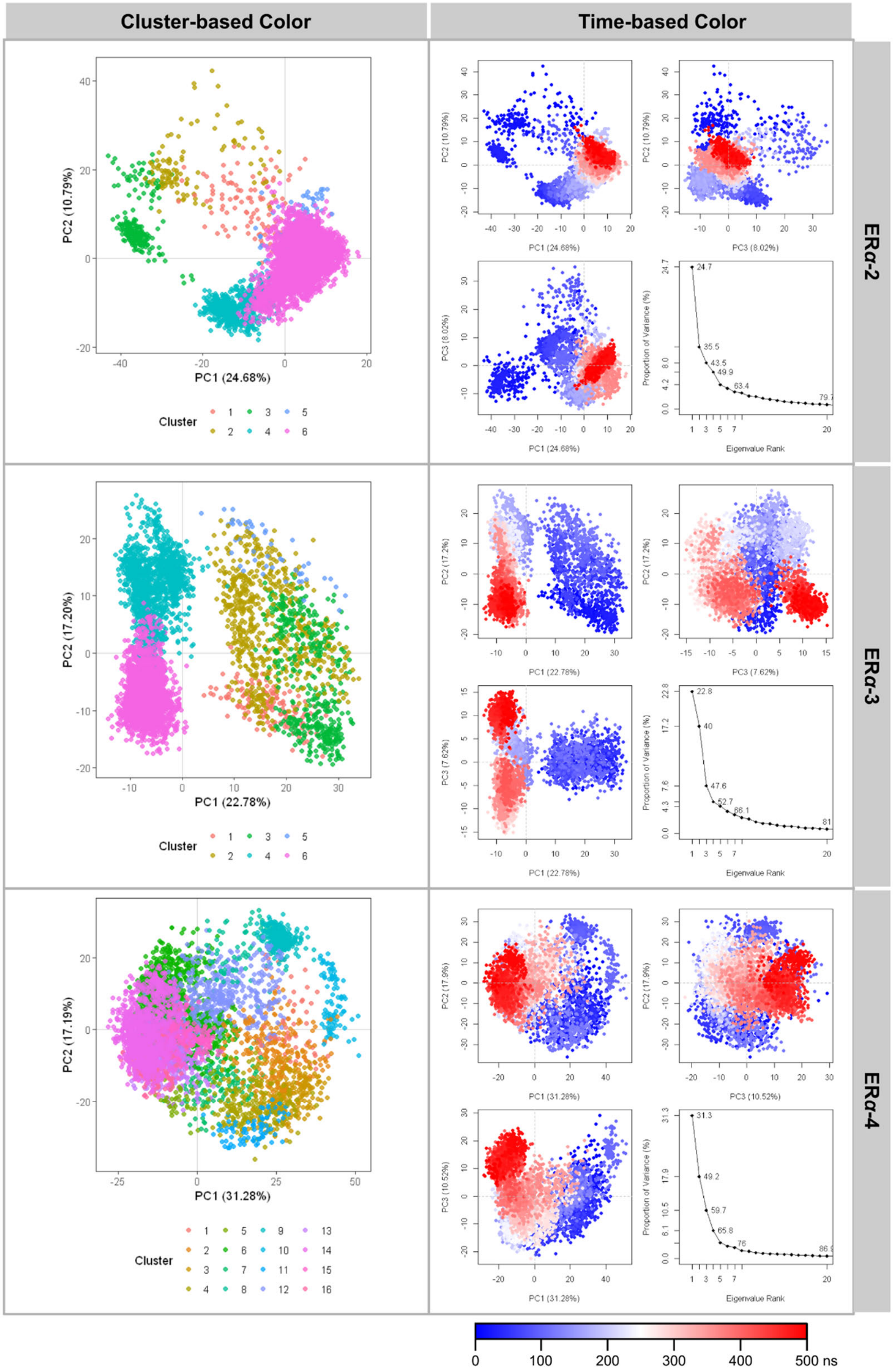

Figure S4. Left panels show principal component (PC) maps colored according to hierarchical clustering on principal component analysis (HCPC). Right panels show PC maps colored according to time scale. Time scale colors are from blue, white, to red.

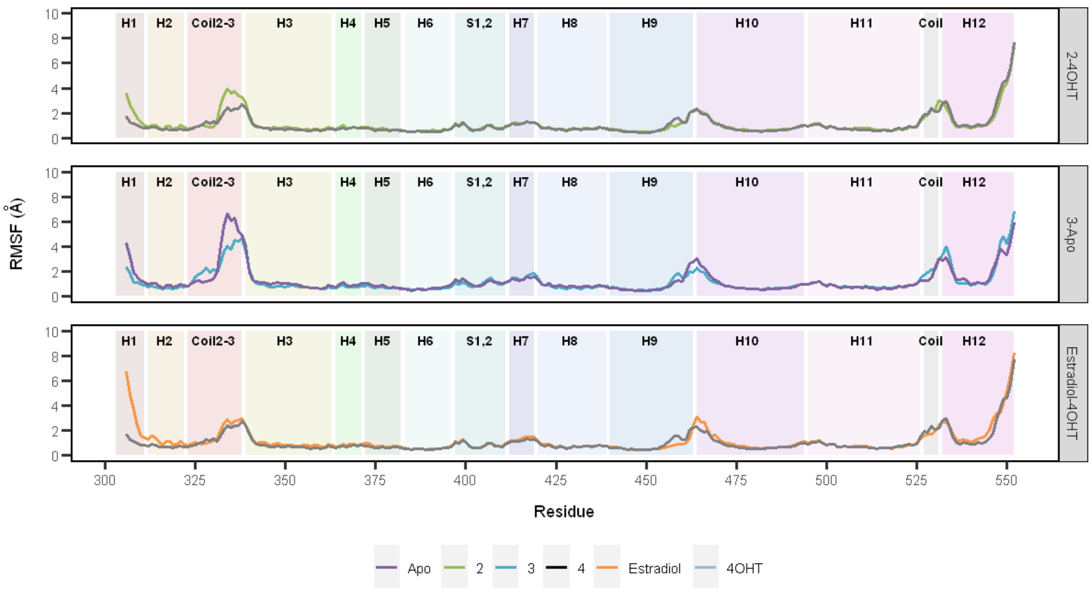

Figure S5. Pair–wise RMSF profile comparation between 2- and 4OHT-bound ERα (top panel), **3**-bound and apo forms of ERα (middle panel), and estradiol- and 4OHT-bound ERα (lower panel).

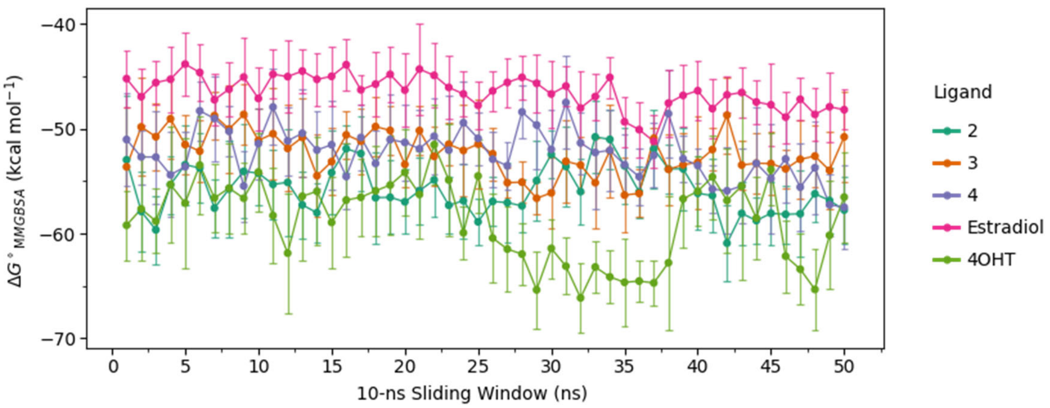

Figure S6. MMGBSA binding energy trajectories of ERα-bound ligands over 500 ns. Each data point was computed from a 10-ns sliding window.

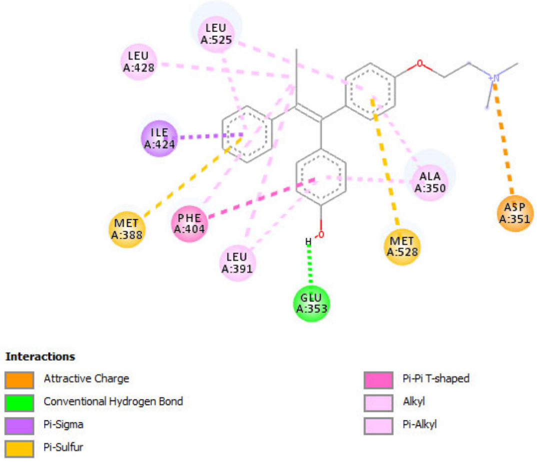

4OHT

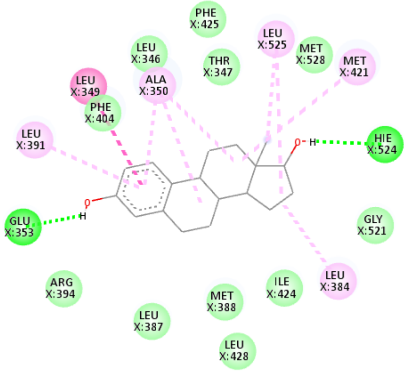

Estradiol

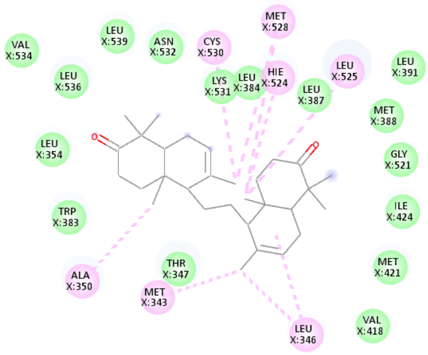

2

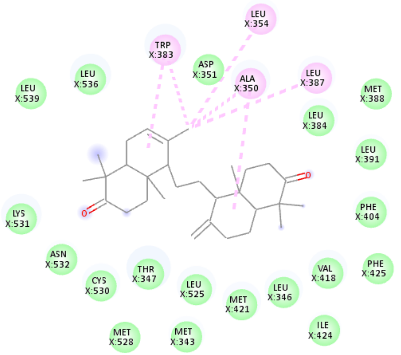

3

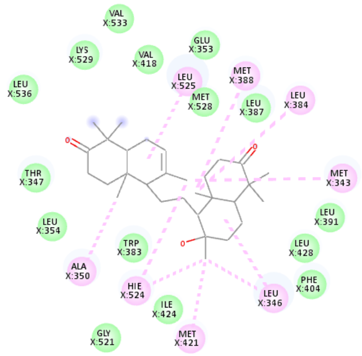

4

Figure S7. Interactions of between ligands and ERα after MD simulations. The ligands include 4OHT, estradiol, **2**, **3**, and **4**. The last frame of each MD system was used for visualization.

Table S1. Re-docking results of 4OHT to ERα. 4OHT and ERα are originated from crystal structure with PDB ID 3ERT.

Cluster	Rank	Sub Rank	Binding Energy (kcal mol^{-1})	Reference RMSD (Å)
1	1	25	–11.50	1.14
1	2	60	–11.49	1.00
1	3	71	–11.48	1.10
1	4	45	–11.47	1.10
1	5	87	–11.46	1.00
1	6	14	–11.45	1.20
1	7	37	–11.44	1.01
1	8	47	–11.43	0.99
1	9	44	–11.43	1.17
1	10	27	–11.43	1.20
1	11	38	–11.42	0.98
1	12	64	–11.42	0.97
1	13	29	–11.42	1.11
1	14	9	–11.41	1.13
1	15	80	–11.41	0.99
1	16	77	–11.40	1.07
1	17	34	–11.40	0.87
1	18	49	–11.39	1.05
1	19	36	–11.39	1.03
1	20	3	–11.39	0.95
1	21	89	–11.39	1.02
1	22	35	–11.38	1.09
1	23	94	–11.38	0.96
1	24	63	–11.38	0.88
1	25	13	–11.38	1.03
1	26	68	–11.38	1.05
1	27	42	–11.38	0.94
1	28	21	–11.38	1.12
1	29	74	–11.37	0.98
1	30	52	–11.37	1.01
1	31	32	–11.37	0.97
1	32	30	–11.36	1.00
1	33	4	–11.36	1.02
1	34	39	–11.36	1.03
1	35	17	–11.36	1.04
1	36	48	–11.35	1.05
1	37	26	–11.34	0.92
1	38	95	–11.32	1.06
1	39	54	–11.31	0.91
1	40	92	–11.31	0.97
1	41	98	–11.31	1.00
1	42	75	–11.31	0.91
1	43	18	–11.31	1.04
1	44	53	–11.31	0.91
1	45	100	–11.31	0.99
1	46	19	–11.31	0.97

Cluster	Rank	Sub Rank	Binding Energy (kcal mol⁻¹)	Reference RMSD (Å)
1	47	2	–11.31	1.01
1	48	65	–11.30	0.92
1	49	59	–11.30	1.10
1	50	93	–11.30	1.00
1	51	43	–11.30	1.03
1	52	12	–11.30	0.89
1	53	91	–11.29	1.07
1	54	1	–11.28	0.98
1	55	51	–11.28	0.94
1	56	84	–11.27	0.86
1	57	40	–11.27	1.05
1	58	10	–11.26	0.90
1	59	86	–11.26	0.97
1	60	85	–11.25	0.99
1	61	73	–11.24	1.03
1	62	76	–11.24	0.90
1	63	15	–11.23	0.97
1	64	81	–11.21	0.96
1	65	69	–11.21	0.89
1	66	96	–11.21	0.93
1	67	99	–11.20	1.00
1	68	61	–11.20	1.06
1	69	7	–11.19	0.97
1	70	6	–11.19	0.90
1	71	88	–11.18	0.96
1	72	62	–11.17	0.95
1	73	22	–11.16	1.05
1	74	58	–11.16	0.94
1	75	5	–11.16	1.09
1	76	11	–11.13	1.02
1	77	33	–11.13	0.81
1	78	66	–11.13	0.92
1	79	31	–11.13	0.81
1	80	50	–11.12	1.05
1	81	70	–11.11	0.76
1	82	82	–11.11	1.31
1	83	67	–11.10	0.68
1	84	78	–11.10	0.95
1	85	8	–11.09	1.08
1	86	24	–11.08	0.96
1	87	41	–11.07	1.20
1	88	83	–11.06	0.83
1	89	90	–11.05	0.82
1	90	46	–11.03	0.96
1	91	23	–11.03	0.66
1	92	72	–11.02	1.02
1	93	16	–10.95	1.12
1	94	79	–10.95	0.92

Cluster	Rank	Sub Rank	Binding Energy (kcal mol⁻¹)	Reference RMSD (Å)
1	95	20	–10.94	0.95
1	96	55	–10.94	0.99
1	97	57	–10.90	0.81
1	98	97	–10.84	0.91
1	99	28	–10.74	0.92
2	1	56	–10.43	3.16

Table S1. Molecular docking results of 4OHT, estradiol, and five onoceranoid triterpenes to ERα.

Ligand	Cluster Rank	Lowest Binding Energy (kcal mol^{-1})	Mean Binding Energy (kcal mol^{-1})	Number in Cluster
**4OHT**	1	–11.50	–11.26	99
	2	–10.43	–10.43	1
**Estradiol**	1	–9.63	–9.62	100
**1**	1	–9.37	–8.57	43
	2	–9.24	–8.42	23
	3	–8.66	–8.12	34
**2**	1	–11.43	–11.08	100
**3**	1	–11.60	–11.49	70
	2	–10.77	–10.55	20
	3	–10.25	–10.25	2
**4**	1	–11.05	–10.86	90
	2	–9.89	–9.31	9
	3	–8.61	–8.61	1
**5**	1	–10.03	–9.71	96
	2	–9.38	–9.38	1
	3	–9.14	–8.93	3

Table S2. Maximum, median, and interquartile range (IQR) of RMSD values of ERα in apo and ligand–binding forms. The RMSD values are calculated from 500-ns MD trajectories.

Complex	RMSD (Å)		
	Maximum Value	Median Value	IQR Value
ERα-**2**	3.77	2.46	0.23
ERα-**3**	3.88	2.66	0.36
ERα-**4**	4.42	3.13	0.67
Apo	4.31	3.28	0.32
ERα-Estradiol	4.28	3.02	0.54
ERα-4OHT	4.05	2.94	0.26

Table S3. Median values of MMGBSA binding energy and its energy terms of **2**, **3**, **4**, estradiol, and 4OHT to ERα.

Ligand	MMGBSA Binding Energy (kcal mol^{-1})	MMGBSA Energy Terms (kcal mol^{-1})			
		vdW	Electrostatics	EGB	ESurf
**2**	–56.10	–58.88	–3.68	13.25	–7.13
**3**	–52.27	–52.57	–7.23	13.97	–6.53
**4**	–52.17	–56.50	–3.67	14.75	–7.03
**Estradiol**	–46.35	–39.20	–19.69	18.01	–5.31
**4OHT**	–57.37	–53.45	–186.71	189.13	–7.55

Table S4. Median values of MMGBSA binding energy contribution and its energy terms of **2**, **3**, **4**, estradiol, and 4OHT to amino acid residues of ERα.

Residue	Median Value of MMGBSA Energy Terms (kcal mol⁻¹)				MMGBSA Energy (kcal mol⁻¹)	Ligand
	Electrostatic	Non–Polar Solvation	Polar Solvation	vdW		
Thr347	–0.238	–1.845	0.218	–2.468	–4.350	2
Leu525	–0.024	–1.879	0.058	–2.399	–4.292	2
Leu346	–0.022	–1.332	–0.156	–2.088	–3.594	2
Met528	–0.067	–1.275	–0.191	–1.852	–3.392	2
Ala350	0.164	–1.345	–0.337	–1.583	–3.089	2
Lys529	–0.255	–0.756	–0.259	–1.166	–2.58	2
Hie524	–0.2	–0.849	0.364	–1.466	–2.232	2
Trp383	0.197	–0.943	–0.2	–1.255	–2.118	2
Asp351	–1.302	–0.792	1.32	–1.187	–1.991	2
Met421	–0.097	–0.706	0.136	–0.991	–1.676	2
Leu354	0.156	–0.72	–0.166	–0.891	–1.617	2
Leu387	–0.175	–0.491	–0.157	–0.702	–1.574	2
Met343	–0.158	–0.699	0.394	–1.051	–1.513	2
Asp426	–0.031	0	0.054	–0.01	0.014	2
Ser395	–0.066	0	0.086	–0.006	0.016	2
Glu330	–0.444	0	0.509	–0.012	0.058	2
Glu419	–0.594	0	0.737	–0.057	0.082	2
Thr347	–0.104	–1.584	0.284	–2.293	–3.687	3
Leu525	0.086	–1.497	–0.18	–1.664	–3.254	3
Ala350	0.12	–1.37	–0.271	–1.543	–3.066	3
Trp383	0.008	–1.434	0.389	–1.846	–2.898	3
Leu387	0.269	–0.962	–0.59	–1.324	–2.599	3
Asn532	–0.625	–0.784	0.225	–0.939	–2.509	3
Leu346	0.041	–0.951	–0.128	–1.43	–2.43	3
Cys530	–0.169	–0.784	0.089	–0.987	–2.012	3
Leu354	0.103	–0.792	–0.286	–0.891	–1.866	3
Met421	–0.058	–0.82	0.03	–1.01	–1.866	3
Leu536	0.077	–0.933	–0.087	–0.92	–1.86	3
Leu384	0.196	–0.739	–0.294	–0.971	–1.841	3
Met388	–0.161	–0.672	0.057	–0.995	–1.751	3
Glu419	–0.472	0	0.505	–0.024	0.017	3
Gly344	–0.043	0	0.157	–0.072	0.033	3
Leu346	0.16	–2.118	–0.283	–2.874	–5.17	4
Leu525	–0.062	–1.952	–0.165	–2.471	–4.658	4
Met528	0.001	–1.202	–0.223	–1.457	–2.885	4
Thr347	–0.096	–1.196	–0.005	–1.512	–2.843	4
Leu387	0.006	–1.004	–0.243	–1.254	–2.533	4
Lys529	–2.232	–0.88	1.658	–1.037	–2.502	4
Met343	–0.202	–1.029	0.337	–1.36	–2.294	4
Met421	–0.105	–0.906	0.068	–1.313	–2.22	4
Met388	–0.072	–0.857	–0.145	–1.137	–2.218	4
Hie524	–0.356	–0.901	0.352	–1.349	–2.206	4
Ala350	0.305	–1.022	–0.213	–1.112	–2.074	4
Ile424	0.076	–0.783	–0.295	–1.023	–2.059	4

Residue	Median Value of MMGBSA Energy Terms (kcal mol⁻¹)				MMGBSA Energy (kcal mol⁻¹)	Ligand
	Electrostatic	Non–Polar Solvation	Polar Solvation	vdW		
Phe404	0.295	–0.901	–0.286	–1.146	–2.049	4
Leu349	0.086	–0.484	–0.445	–0.928	–1.672	4
Leu384	0.005	–0.703	–0.087	–0.869	–1.651	4
Leu391	–0.021	–0.799	–0.056	–0.748	–1.651	4
Asp426	–0.317	0	0.361	–0.017	0.019	4
Ser518	–0.052	0	0.168	–0.078	0.038	4
Glu385	0.128	0	–0.053	–0.051	0.04	4
Glu353	–7.578	–0.552	2.539	0.677	–4.896	Estradiol
Leu391	–0.256	–1.101	–0.408	–1.4	–3.116	Estradiol
Hie524	–2.543	–0.814	1.041	–0.71	–3.037	Estradiol
Leu387	0.125	–1.056	–0.571	–1.511	–2.981	Estradiol
Leu525	–0.27	–1.058	–0.437	–1.19	–2.943	Estradiol
Leu346	–0.174	–1.057	–0.091	–1.548	–2.757	Estradiol
Met388	0.256	–1.075	–0.27	–1.491	–2.571	Estradiol
Ala350	–0.085	–0.87	–0.276	–0.956	–2.206	Estradiol
Phe404	–0.34	–0.951	0.441	–1.344	–2.174	Estradiol
Leu384	0.01	–0.872	0.007	–1.107	–1.957	Estradiol
Met421	–0.065	–0.813	0.167	–1.146	–1.867	Estradiol
Ile424	0.159	–0.715	–0.326	–0.901	–1.787	Estradiol
Leu349	0.015	–0.496	–0.361	–0.744	–1.578	Estradiol
Glu330	0.002	0	0.03	–0.007	0.027	Estradiol
Gly344	0.037	0	0.014	–0.028	0.027	Estradiol
Glu385	0.069	0	0.009	–0.059	0.028	Estradiol
Asp426	–0.214	0	0.26	–0.014	0.03	Estradiol
Glu419	–0.367	0	0.439	–0.029	0.032	Estradiol
Ser395	–0.028	0	0.15	–0.018	0.099	Estradiol
Asp351	0.584	0	–0.399	–0.074	0.114	Estradiol
Tyr526	0.057	0	0.172	–0.069	0.154	Estradiol
Asp351	–37.689	–0.791	33.634	–0.844	–6.811	4OHT
Glu353	–23.177	–0.649	17.182	0.434	–6.092	4OHT
Leu525	–0.412	–1.896	–0.043	–2.433	–4.74	4OHT
Ala350	0.186	–1.613	–0.059	–2.132	–3.793	4OHT
Leu387	0.365	–1.05	–0.688	–1.4	–2.794	4OHT
Ile424	0.173	–0.95	–0.644	–1.225	–2.626	4OHT
Leu346	–0.451	–1.096	0.649	–1.724	–2.548	4OHT
Leu391	0.624	–0.936	–0.991	–1.191	–2.507	4OHT
Thr347	–2.612	–0.903	2.48	–1.394	–2.44	4OHT
Phe404	–0.502	–1.092	0.551	–1.295	–2.335	4OHT
Met528	0.459	–0.928	–0.62	–1.192	–2.289	4OHT
Leu349	–0.341	–0.616	–0.367	–0.915	–2.238	4OHT
Met388	0.285	–0.789	–0.406	–0.996	–1.881	4OHT
Cys530	–0.466	–0.694	0.033	–0.709	–1.821	4OHT
Leu384	–0.114	–0.774	0.07	–0.992	–1.813	4OHT
Leu541	0.194	0	–0.173	–0.002	0.017	4OHT
Asp426	–7.412	0	7.458	–0.016	0.025	4OHT
Lys416	7.007	0	–6.963	–0.011	0.035	4OHT

Residue	Median Value of MMGBSA Energy Terms (kcal mol⁻¹)				MMGBSA Energy (kcal mol⁻¹)	Ligand
	Electrostatic	Non–Polar Solvation	Polar Solvation	vdW		
Ala405	0.03	0	0.052	–0.038	0.042	4OHT
Tyr526	–0.209	0	0.344	–0.063	0.067	4OHT
Ser395	0.229	0	–0.125	–0.016	0.084	4OHT

Table S5. Conventional H-bond occurrence between ligands and ERα over 500-ns MD trajectories.

Ligand	ERa	Number of Frames	Fraction	Percentage (%)
2	Trp383@HE1	23	0.0005	0.05
2	Lys529@HZ3	10	0.0002	0.02
2	Lys531@HZ3	10	0.0002	0.02
2	Lys531@HZ2	6	0.0001	0.01
2	Lys529@HZ1	5	0.0001	0.01
2	Lys529@HZ2	4	0.0001	0.01
2	Lys531@HZ1	2	0	0
2	Asn532@HD21	1	0	0
3	Asn532@H	2773	0.0555	5.55
3	Asn532@HD22	1862	0.0372	3.72
3	Val533@H	1443	0.0289	2.89
3	Lys531@H	244	0.0049	0.49
3	Asn532@HD21	241	0.0048	0.48
3	Cys530@H	108	0.0022	0.22
3	Val534@H	107	0.0021	0.21
3	Lys531@HZ3	20	0.0004	0.04
3	Lys531@HZ2	13	0.0003	0.03
3	Lys529@HZ2	12	0.0002	0.02
3	Lys531@HZ1	11	0.0002	0.02
3	Lys529@HZ3	10	0.0002	0.02
3	Lys529@HZ1	9	0.0002	0.02
4	Gly521@O	1082	0.0216	2.16
4	His524@ND1	804	0.0161	1.61
4	Lys529@HZ1	205	0.0041	0.41
4	Lys529@HZ3	174	0.0035	0.35
4	Lys529@HZ2	173	0.0035	0.35
4	Thr347@HG1	14	0.0003	0.03
4	Gly420@O	8	0.0002	0.02
4	Cys530@H	7	0.0001	0.01
4	His524@O	1	0	0
4	His524@HE2	1	0	0
Estradiol	His524@ND1	20156	0.4031	40.31
Estradiol	Glu353@OE1	19556	0.3911	39.11
Estradiol	Glu353@OE2	15848	0.317	31.7
Estradiol	Arg394@HH22	271	0.0054	0.54
Estradiol	His524@O	88	0.0018	0.18
Estradiol	Gly521@O	64	0.0013	0.13
Estradiol	Leu387@O	14	0.0003	0.03
Estradiol	Thr347@OG1	7	0.0001	0.01

Ligand	ERa	Number of Frames	Fraction	Percentage (%)
Estradiol	His524@HE2	6	0.0001	0.01
Estradiol	Glu419@O	2	0	0
Estradiol	Arg394@HH12	2	0	0
Estradiol	Phe404@O	1	0	0
Estradiol	Arg394@NH2	1	0	0
4OHT	Glu353@OE2	30772	0.6154	61.54
4OHT	Asp351@OD1	10707	0.2141	21.41
4OHT	Asp351@OD2	8589	0.1718	17.18
4OHT	Glu353@OE1	615	0.0123	1.23
4OHT	Arg394@HH22	43	0.0009	0.09
4OHT	Lys531@O	34	0.0007	0.07
4OHT	Thr347@OG1	19	0.0004	0.04
4OHT	Thr347@O	4	0.0001	0.01
4OHT	Leu387@O	2	0	0
4OHT	Ala350@O	1	0	0

Table S6. Contact surface area (CSA) between **2** and ERα residues every 50 ns over a 500-ns MD trajectory.

Residue	Contact Surface Area (Å^2)										
	0 ns	50 ns	100 ns	150 ns	200 ns	250 ns	300 ns	350 ns	400 ns	450 ns	500 ns
Met342	0.00	0.42	0.00	0.12	0.00	1.95	0.00	0.06	0.00	0.00	0.00
Met343	27.63	20.93	26.50	17.28	28.96	26.33	14.90	27.93	20.07	27.70	24.74
Gly344	0.00	0.14	0.15	0.87	0.00	0.54	0.00	0.17	0.00	0.00	0.00
Leu345	0.00	0.00	0.00	0.00	0.00	0.00	0.00	0.00	0.00	0.02	0.11
Leu346	0.00	34.86	34.91	37.61	34.27	40.16	24.71	34.73	42.15	32.72	37.70
Thr347	26.42	45.34	45.48	47.88	43.89	46.49	46.38	48.20	24.95	49.74	43.05
Asn348	0.00	0.51	1.51	0.97	2.34	1.88	0.00	0.71	0.00	0.22	0.27
Leu349	0.97	0.00	0.11	0.49	0.00	0.24	0.23	0.00	21.68	0.00	0.00
Ala350	38.70	30.12	35.39	33.23	31.42	29.83	29.65	29.95	27.61	28.66	24.12
Asp351	27.13	33.91	32.92	29.78	32.46	28.48	34.80	23.67	0.00	21.34	6.28
Arg352	0.00	0.00	0.00	0.15	0.00	0.00	0.21	0.00	0.00	0.00	0.00
Glu353	15.32	0.00	2.25	0.00	0.00	0.00	0.00	0.00	4.07	0.00	0.00
Leu354	21.21	26.12	22.60	19.87	27.34	24.98	30.11	21.26	22.55	22.11	23.32
Trp383	30.60	37.21	29.20	40.00	32.11	33.03	34.08	29.26	37.85	34.85	32.87
Leu384	24.36	20.86	20.55	22.71	16.58	13.71	22.24	26.06	28.97	22.68	16.75
Leu387	42.95	25.99	18.39	13.56	25.51	18.55	8.29	0.69	29.16	0.45	20.77
Met388	27.29	26.54	21.05	12.81	15.34	21.02	15.87	13.92	25.48	20.63	17.39
Leu391	17.24	17.50	17.70	5.57	13.52	16.70	3.33	0.00	18.06	0.00	9.45
Arg394	0.52	0.00	0.00	0.00	0.00	0.00	0.00	0.00	0.00	0.00	0.00
Phe404	13.20	18.55	15.08	14.19	17.34	7.75	0.00	3.28	19.91	0.00	10.05
Cys417	0.00	0.00	0.00	0.00	0.00	0.00	0.00	0.00	0.00	0.67	0.00
Val418	0.00	8.21	7.26	9.77	11.94	9.53	9.63	9.62	0.88	5.63	3.58
Glu419	3.19	0.00	0.00	0.00	0.00	0.00	0.00	0.00	0.00	0.00	0.00
Gly420	15.69	0.00	0.00	0.00	0.00	0.00	0.00	0.00	0.00	0.00	0.00
Met421	43.83	16.45	14.90	26.78	25.29	17.08	22.36	26.67	19.54	29.17	21.35
Ile424	17.88	14.40	14.06	18.28	19.74	21.94	25.12	24.63	18.78	19.43	23.80
Phe425	0.00	0.00	0.00	0.00	0.00	2.72	9.02	0.00	0.00	0.00	1.05
Met427	0.00	0.00	0.00	0.00	0.00	0.73	0.00	0.00	0.00	0.00	0.00

Residue	Contact Surface Area (Å²)										
	0 ns	50 ns	100 ns	150 ns	200 ns	250 ns	300 ns	350 ns	400 ns	450 ns	500 ns
Leu428	12.93	12.54	7.57	5.74	10.78	8.02	9.22	1.79	13.71	0.00	2.69
Met517	0.00	0.00	0.00	0.00	0.00	0.00	0.00	0.00	0.80	1.32	0.00
Ser518	0.00	0.00	0.00	0.00	0.00	0.00	0.00	0.00	0.19	0.00	0.00
Lys520	0.00	0.00	0.00	0.00	0.00	0.00	0.57	0.04	0.91	0.57	0.00
Gly521	3.43	9.67	10.66	12.44	2.68	10.92	13.35	16.53	14.62	14.90	15.04
Met522	0.00	0.00	0.00	1.45	0.00	0.00	1.65	2.65	11.22	3.64	2.39
Leu525	34.17	31.32	31.88	35.26	29.82	33.49	33.34	36.81	44.00	41.21	41.36
Met528	23.40	39.82	31.05	38.09	34.94	31.29	38.05	30.18	7.60	24.95	20.80
Lys529	0.00	45.06	38.95	35.73	40.54	47.06	0.00	0.47	0.00	0.00	0.00
Cys530	29.32	0.65	13.55	1.14	0.38	2.02	35.63	30.29	2.41	10.85	26.24
Lys531	0.00	0.00	0.00	0.00	0.00	0.00	0.42	6.39	33.70	26.35	28.42
Asn532	0.00	0.00	0.00	0.00	28.39	0.00	27.25	37.25	32.63	31.76	29.72
Val533	28.18	17.92	16.57	31.76	0.00	22.60	0.00	0.00	0.60	0.20	0.56
Val534	8.84	2.08	5.76	14.33	3.47	2.32	0.00	0.28	4.57	7.49	6.68
Pro535	0.00	0.00	0.13	4.25	0.00	0.00	0.00	0.00	3.17	1.52	3.44
Leu536	15.39	25.48	20.79	30.74	18.00	23.64	19.94	23.12	40.06	34.26	35.40
Leu539	14.60	21.30	23.61	11.62	25.84	20.49	18.17	24.45	17.10	20.86	21.59
Met543	0.00	1.68	0.00	0.00	0.00	0.00	0.34	0.00	0.00	0.82	0.00

Table S7. Contact surface area (CSA) between **3** and ERα residues every 50 ns over a 500-ns MD trajectory.

Residue	Contact Surface Area (Å²)										
	0 ns	50 ns	100 ns	150 ns	200 ns	250 ns	300 ns	350 ns	400 ns	450 ns	500 ns
Met343	34.11	17.83	17.91	11.34	37.35	27.67	10.25	15.52	21.15	18.88	26.35
Leu346	21.83	26.41	16.02	15.44	31.23	36.08	26.36	22.80	30.71	36.08	35.08
Thr347	29.82	43.96	43.63	41.91	45.33	42.31	39.72	43.30	47.76	42.43	45.76
Asn348	0.00	0.07	0.04	0.00	0.30	0.00	0.00	0.00	0.22	0.00	0.16
Ala350	32.18	29.96	31.71	31.41	27.68	31.50	31.74	30.71	32.35	26.89	27.60
Asp351	2.37	23.10	19.13	29.00	20.25	16.83	16.50	15.17	19.56	16.52	13.16
Arg352	0.00	0.24	0.00	0.00	0.00	0.00	0.14	0.00	0.00	0.00	0.00
Glu353	0.00	0.00	0.37	0.10	0.00	0.00	0.00	0.36	0.00	0.06	0.00
Leu354	19.18	22.16	38.20	36.23	19.61	25.92	28.13	20.62	29.33	33.57	26.45
Trp383	36.29	31.72	37.28	37.36	43.21	41.88	42.11	37.98	37.27	35.40	40.75
Leu384	25.35	18.83	22.61	24.60	22.62	20.50	23.38	24.21	18.92	22.73	24.36
Leu387	22.93	35.91	15.43	15.86	30.17	30.35	32.24	33.21	43.44	33.85	33.51
Met388	17.06	31.18	14.28	11.74	24.79	25.17	29.55	25.94	30.53	25.16	8.46
Leu391	4.38	14.51	2.54	0.00	20.50	23.68	21.99	17.41	13.81	15.57	16.70
Arg394	0.00	0.00	0.00	0.00	0.00	1.75	0.00	0.00	0.00	0.00	0.00
Leu402	0.00	0.65	0.00	0.00	0.00	0.00	0.00	0.00	0.00	0.00	0.00
Phe404	0.72	22.04	4.51	0.00	20.44	21.15	18.28	23.19	14.54	18.93	20.98
Val418	0.00	0.00	0.00	0.00	0.00	0.00	0.00	0.00	0.00	0.00	22.43
Glu419	1.79	0.00	0.00	0.00	0.00	0.00	0.00	0.00	0.00	0.00	0.00
Gly420	8.09	0.00	0.00	0.00	0.00	0.00	0.00	0.00	0.00	0.00	0.00
Met421	29.97	27.88	27.20	32.05	17.83	24.88	20.90	26.02	29.86	23.43	29.42
Ile424	17.63	19.55	21.67	22.71	16.21	2.27	24.35	16.82	9.29	18.40	12.36
Phe425	0.00	10.05	13.08	5.18	0.00	0.00	0.14	0.00	0.00	12.92	13.31
Leu428	4.00	19.70	11.89	4.57	11.02	8.33	14.77	14.01	14.29	15.02	7.97

Residue	Contact Surface Area (Å²)										
	0 ns	50 ns	100 ns	150 ns	200 ns	250 ns	300 ns	350 ns	400 ns	450 ns	500 ns
Met517	0.68	0.00	0.00	0.00	0.00	0.00	0.00	0.00	0.00	0.00	0.00
Ser518	0.20	0.00	0.00	0.00	0.00	0.00	0.00	0.00	0.00	0.00	0.00
Lys520	0.44	0.00	0.00	0.00	0.00	0.00	0.00	0.00	0.00	0.00	0.00
Gly521	17.17	0.00	15.71	10.71	2.43	6.68	5.07	1.33	0.86	5.20	0.85
Met522	4.36	0.00	3.99	5.30	0.00	0.00	0.06	0.00	0.00	0.00	0.00
Leu525	38.07	37.02	43.94	36.29	46.02	42.40	37.56	37.21	36.75	37.09	32.30
Met528	41.20	12.77	12.19	6.30	0.00	0.00	16.19	28.91	13.42	11.65	9.63
Lys529	26.29	2.14	0.00	0.00	0.00	0.00	0.00	0.00	0.00	0.00	0.00
Cys530	2.47	33.41	0.00	45.01	31.96	0.00	40.58	26.95	30.98	26.37	18.43
Lys531	0.00	22.82	0.00	40.57	19.65	11.45	17.45	13.52	8.18	39.82	40.77
Asn532	0.00	23.10	38.29	25.51	29.31	29.34	29.53	29.66	29.83	24.19	20.17
Val533	25.66	0.00	0.00	0.00	0.00	27.04	0.00	18.66	5.70	0.00	0.00
Val534	7.66	0.00	0.00	0.47	0.00	21.37	6.20	24.14	4.17	0.00	0.10
Pro535	0.24	0.00	0.00	0.00	0.00	3.51	4.94	4.05	0.00	0.00	0.00
Leu536	29.58	32.86	25.15	31.53	28.08	34.56	34.46	36.55	24.09	27.98	30.60
Leu539	25.06	17.54	30.56	19.81	4.73	19.64	12.17	19.37	23.65	24.68	11.61
Met543	0.00	0.00	9.31	3.94	0.00	0.00	0.00	0.00	0.00	0.00	0.00

Table S8. Contact surface area (CSA) between **4** and ER*α* residues every 50 ns over a 500-ns MD trajectory.

Residue	Contact Surface Area (A2)										
	0 ns	50 ns	100 ns	150 ns	200 ns	250 ns	300 ns	350 ns	400 ns	450 ns	500 ns
Met342	0.00	0.00	0.00	0.46	0.00	0.00	0.00	0.00	0.00	0.01	0.00
Met343	27.54	36.13	26.85	32.10	31.66	34.19	39.61	29.37	36.53	38.21	23.85
Gly344	0.00	0.00	0.00	0.00	0.00	0.00	0.00	0.00	0.62	0.23	0.00
Leu346	32.19	48.75	48.76	45.63	46.84	48.56	49.19	46.15	45.61	48.31	46.36
Thr347	48.39	39.57	35.44	37.92	35.67	38.55	40.86	36.82	28.83	39.92	37.62
Asn348	0.47	0.00	0.00	0.00	0.00	0.45	0.00	0.09	0.00	0.00	0.00
Leu349	2.55	17.41	20.69	20.95	19.11	13.62	7.12	23.70	28.10	12.53	0.00
Ala350	28.46	24.16	32.53	32.37	34.55	34.45	16.19	35.07	26.62	27.54	27.76
Asp351	28.76	0.00	0.00	0.00	1.43	0.00	0.00	0.13	0.38	0.00	0.00
Glu353	0.00	0.12	16.64	24.47	25.54	13.51	0.00	13.00	28.66	4.15	15.59
Leu354	15.14	0.00	0.00	5.45	3.30	0.00	0.00	0.00	0.26	0.00	12.35
Trp383	36.94	29.90	3.54	20.15	16.80	8.04	5.75	19.54	3.38	11.55	33.12
Leu384	23.27	20.61	18.78	20.30	14.74	33.19	18.15	17.91	24.48	26.28	19.76
Glu385	0.00	0.00	0.00	0.00	0.00	1.06	0.00	0.00	0.00	0.00	0.00
Leu387	27.14	29.43	28.12	37.21	40.66	35.55	19.59	31.21	42.98	28.81	35.95
Met388	19.70	28.69	28.91	31.72	34.53	34.49	18.21	28.82	31.58	30.63	33.03
Leu391	7.05	15.96	19.61	26.86	24.68	16.90	7.91	19.55	24.87	22.71	26.99
Arg394	0.00	0.00	0.38	5.68	7.35	0.24	0.00	1.08	8.97	0.00	4.17
Leu402	0.00	0.00	0.00	1.19	0.00	0.00	0.00	0.00	0.00	0.00	0.00
Phe404	16.55	18.11	31.12	28.66	27.34	8.74	10.36	45.31	40.29	23.44	41.22
Ala405	0.00	0.00	0.00	0.00	0.00	0.00	0.00	0.00	0.38	0.00	0.00
Val418	0.00	11.89	5.97	3.28	5.73	17.24	29.07	13.82	13.53	2.85	6.83
Glu419	0.00	4.05	0.00	0.00	0.00	0.00	19.86	0.00	0.00	15.98	0.00
Gly420	0.00	3.25	0.00	0.00	0.00	0.31	8.79	0.00	0.00	0.63	0.00
Met421	26.91	33.92	24.03	25.34	18.05	26.87	38.82	23.39	25.43	41.78	25.38

Residue	Contact Surface Area (A2)										
	0 ns	50 ns	100 ns	150 ns	200 ns	250 ns	300 ns	350 ns	400 ns	450 ns	500 ns
Ile424	16.43	30.49	20.45	13.87	16.33	26.87	27.69	19.46	22.33	22.30	18.14
Phe425	0.00	0.18	0.00	0.00	0.00	0.00	0.00	0.00	0.54	0.00	0.00
Leu428	4.13	14.75	8.69	19.31	7.52	6.75	2.86	11.46	23.18	22.47	21.55
Met517	0.00	0.01	0.00	0.00	0.00	0.00	0.00	0.00	0.00	0.00	0.00
Lys520	0.31	1.38	0.36	0.00	0.00	0.68	1.12	0.00	0.00	1.18	0.00
Gly521	16.67	16.32	14.88	2.01	3.51	17.06	19.72	7.49	11.41	16.50	7.63
Met522	7.54	2.47	1.70	0.00	0.00	1.97	2.73	0.00	1.77	6.14	0.00
Leu525	45.03	35.69	46.09	43.46	40.57	36.95	39.62	40.74	32.88	48.84	38.20
Tyr526	0.00	0.00	0.00	0.54	0.02	0.00	0.00	0.00	0.01	0.00	0.00
Met528	28.43	35.74	44.75	22.03	20.47	40.88	43.97	32.36	32.41	28.34	18.76
Lys529	2.81	0.00	22.36	31.75	27.05	32.05	0.00	32.86	12.24	45.55	27.65
Cys530	0.00	0.00	0.00	1.62	6.76	0.00	0.00	2.84	0.00	1.71	0.00
Val533	0.00	0.00	4.14	33.37	30.68	0.00	0.00	28.73	0.00	0.00	14.43
Leu536	27.82	0.00	0.00	0.00	0.00	0.00	0.00	0.00	0.00	0.00	17.70
Leu539	14.63	0.00	1.23	0.00	0.00	0.00	0.00	0.00	0.00	0.00	0.00

Table S9. Contact surface area (CSA) between estradiol and ERα residues every 50 ns over a 500-ns MD trajectory.

Residue	Contact Surface Area (A2)										
	0 ns	50 ns	100 ns	150 ns	200 ns	250 ns	300 ns	350 ns	400 ns	450 ns	500 ns
Met343	20.96	0.00	0.00	25.31	0.00	0.00	13.10	0.00	0.00	0.00	0.49
Leu345	0.00	0.00	0.00	0.00	0.00	0.00	0.00	0.42	0.00	0.00	0.00
Leu346	30.08	31.82	32.18	34.67	29.82	31.04	24.90	37.59	34.25	32.41	29.45
Thr347	11.01	10.04	8.33	9.84	8.02	1.32	14.93	19.74	12.91	21.39	23.86
Asn348	0.00	0.00	0.00	0.00	0.00	0.00	0.00	0.00	0.00	0.00	0.31
Leu349	16.98	15.49	10.14	18.32	21.61	20.09	16.56	30.41	26.74	16.04	28.16
Ala350	21.60	24.68	28.68	23.89	23.94	25.67	21.94	31.92	28.69	27.78	28.47
Asp351	0.00	0.00	0.00	0.00	0.00	0.00	0.00	0.00	0.00	0.00	0.06
Glu353	18.11	17.95	28.31	18.38	22.61	26.53	23.77	24.64	20.98	20.27	24.38
Trp383	11.83	9.83	1.26	7.16	1.00	3.33	1.99	2.69	0.00	9.53	0.00
Leu384	21.66	19.15	27.87	30.60	19.90	22.71	16.75	23.79	26.52	26.61	23.48
Leu387	42.72	38.72	30.83	32.36	37.47	35.68	41.80	26.00	29.38	34.00	20.74
Met388	30.46	27.56	34.24	30.09	29.27	33.82	31.63	22.06	28.69	31.49	25.21
Gly390	2.20	1.19	1.83	0.72	0.60	0.60	1.55	0.00	0.00	0.00	0.00
Leu391	28.38	25.71	31.36	28.41	28.81	27.85	29.60	25.56	26.02	30.31	25.00
Arg394	15.53	14.56	12.73	13.32	10.15	13.55	16.85	10.16	11.04	17.67	8.23
Leu402	0.00	0.00	0.00	0.00	0.10	0.00	0.00	0.00	0.00	0.00	0.00
Phe404	21.17	30.06	26.27	19.86	23.71	27.12	29.17	34.36	29.68	31.09	35.62
Leu408	0.00	0.00	0.00	0.00	0.00	0.00	0.00	0.63	0.00	0.00	0.00
Val418	0.00	0.71	2.05	1.05	0.05	0.00	0.00	0.00	0.00	0.94	0.00
Glu419	0.24	0.00	0.00	0.00	0.00	0.00	0.00	0.00	0.00	0.00	0.00
Gly420	0.74	0.00	0.00	0.00	0.00	0.00	0.00	0.00	0.00	0.00	0.00
Met421	28.11	25.60	18.69	22.87	22.66	17.62	21.80	13.21	14.19	20.09	22.46
Ile424	13.04	19.39	17.65	13.13	22.72	18.15	21.85	7.69	23.32	12.44	18.15
Phe425	0.00	0.00	0.00	0.00	0.00	0.00	0.00	0.00	7.55	0.00	3.49
Met427	0.00	0.00	0.00	0.00	0.00	0.00	0.00	0.00	1.62	0.00	0.00
Leu428	11.21	15.16	20.04	14.67	20.54	19.80	19.87	7.85	9.69	12.29	5.26

Residue	Contact Surface Area (A2)										
	0 ns	50 ns	100 ns	150 ns	200 ns	250 ns	300 ns	350 ns	400 ns	450 ns	500 ns
Met517	0.00	0.00	0.00	0.00	0.00	0.49	0.00	0.00	0.00	0.00	0.00
Lys520	0.00	0.54	0.69	0.00	0.51	0.72	0.28	0.00	0.31	0.08	0.10
Gly521	13.81	13.56	14.71	11.39	15.10	14.46	12.80	12.16	13.75	13.09	13.31
Met522	0.71	2.03	3.71	0.00	1.47	1.31	0.98	2.47	3.68	2.12	3.02
Leu525	27.90	27.62	32.41	32.22	26.67	24.68	29.81	29.53	26.78	24.37	30.52
Tyr526	0.00	0.00	0.00	0.00	0.00	0.00	0.02	0.00	0.00	0.00	0.00
Met528	4.56	7.53	8.93	3.09	20.96	16.81	0.00	16.02	15.60	8.33	12.05

Table S10. Contact surface area (CSA) between 4OHT and ERα residues every 50 ns over a 500-ns MD trajectory.

Residue	Contact Surface Area (A2)										
	0 ns	50 ns	100 ns	150 ns	200 ns	250 ns	300 ns	350 ns	400 ns	450 ns	500 ns
Leu327	0.00	1.88	1.58	0.00	2.11	3.75	0.31	2.01	3.29	0.00	0.00
Met343	29.42	19.57	0.00	14.87	0.00	0.00	0.00	0.00	0.00	0.00	26.07
Leu345	1.26	0.00	0.00	0.00	0.00	0.00	0.00	0.00	0.00	0.00	0.00
Leu346	40.90	33.56	31.20	33.60	39.88	29.39	32.16	35.63	35.67	35.90	36.91
Thr347	39.12	3.16	12.33	35.14	29.25	22.46	29.84	29.01	22.37	36.34	43.66
Asn348	0.00	0.00	0.00	0.00	0.00	0.00	0.68	0.34	1.19	2.84	0.09
Leu349	25.37	26.38	23.76	25.63	27.36	22.46	24.06	24.61	20.65	24.92	25.37
Ala350	25.78	34.56	33.40	26.25	28.93	31.92	32.08	32.53	27.26	29.93	26.70
Asp351	29.39	19.92	18.99	31.36	6.35	19.41	28.69	26.37	12.89	34.56	0.00
Glu353	20.25	26.73	25.11	21.15	22.57	20.77	22.64	23.25	22.41	22.48	25.63
Leu354	5.61	22.71	21.41	8.48	0.00	17.55	17.76	20.79	0.00	0.00	0.00
Trp383	29.75	30.42	32.54	23.59	28.09	37.64	30.10	23.70	34.97	23.19	22.59
Leu384	24.72	20.21	21.13	27.61	20.61	20.05	21.34	22.56	21.48	19.93	26.83
Leu387	30.02	22.87	28.91	28.48	30.91	33.10	29.97	29.56	33.51	35.75	28.62
Met388	28.51	16.45	30.58	31.09	27.58	25.27	27.33	21.51	23.72	21.95	31.34
Leu391	25.41	29.08	23.73	23.39	23.84	25.15	22.77	25.73	25.38	22.94	25.66
Arg394	10.35	10.48	7.24	8.00	8.39	9.26	6.89	7.43	9.01	4.35	9.36
Leu402	0.00	0.00	1.71	0.00	3.46	1.27	0.44	0.00	3.39	3.15	0.00
Phe404	33.74	36.32	40.77	33.55	37.43	40.64	34.79	32.39	37.95	34.10	33.83
Val418	1.13	0.27	0.00	0.00	0.00	0.00	0.00	0.00	0.00	0.26	0.00
Glu419	4.75	0.00	0.00	0.00	0.00	0.00	0.00	0.00	0.00	0.00	0.00
Gly420	6.62	0.00	0.00	0.00	0.00	0.00	0.00	0.00	0.00	0.00	0.00
Met421	32.76	25.34	25.71	19.22	26.44	22.89	25.11	21.93	22.71	22.90	17.26
Ile424	17.53	28.21	26.48	19.07	25.87	25.45	28.98	28.45	29.14	26.07	27.92
Phe425	0.00	18.07	8.28	1.09	9.28	4.40	16.40	20.11	20.01	18.35	11.65
Leu428	15.48	17.31	17.22	21.98	15.93	21.73	13.81	15.81	13.33	16.19	21.46
Met517	0.00	0.00	1.44	0.07	1.29	2.91	0.74	0.66	0.03	0.00	2.03
Ser518	0.00	0.00	0.00	0.00	0.49	0.00	0.00	0.00	0.00	0.00	0.00
Lys520	0.54	0.00	1.64	0.29	1.04	0.45	1.17	0.63	0.52	0.00	0.00
Gly521	13.68	8.82	12.89	12.91	13.04	12.66	12.96	12.91	11.88	11.65	11.87
Met522	2.37	1.77	2.05	4.11	4.22	2.26	2.55	1.75	6.03	0.47	1.86
Leu525	36.83	38.11	37.95	37.98	43.96	38.87	38.67	39.69	42.76	36.54	54.69
Ser527	0.00	0.00	1.17	0.00	0.00	0.00	2.45	0.00	3.36	0.00	0.00
Met528	9.30	15.57	35.60	16.58	28.80	30.83	29.21	29.66	35.92	33.43	1.97
Cys530	0.00	20.44	32.78	30.52	25.25	37.22	28.53	32.26	24.20	29.38	30.75

Residue	Contact Surface Area (A2)										
	0 ns	50 ns	100 ns	150 ns	200 ns	250 ns	300 ns	350 ns	400 ns	450 ns	500 ns
Lys531	0.00	0.00	1.37	1.99	13.41	12.56	6.35	2.88	13.73	5.13	14.55
Asn532	0.00	26.33	0.71	0.00	23.86	0.43	0.00	0.00	12.00	0.00	22.49
Val533	0.00	0.00	0.00	0.00	7.91	0.00	0.00	0.00	0.00	0.00	4.98
Val534	0.00	2.69	3.67	10.08	19.41	0.46	1.38	0.00	28.40	0.00	24.67
Leu536	0.00	25.35	26.96	0.00	0.00	24.01	2.86	3.70	3.29	0.91	0.00
Leu539	0.00	5.56	17.40	0.00	0.00	3.03	2.81	0.00	0.00	0.00	0.00
